# Supplementary material for: Changes in social norms during the early stages of the COVID-19 pandemic across 43 countries
Source: Nat Commun. 2024 Feb 16;15:1436. doi: 10.1038/s41467-024-44999-5 (PMC10873354; doi:10.1038/s41467-024-44999-5)
Supplement: Supplementary file 1 — Supplementary Information [file 41467_2024_44999_MOESM1_ESM.pdf]

Supplementary Materials for

Changes in Social Norms During the Early Stages of the COVID-19 Pandemic Across 43 Countries

Authors: Giulia Andrighetto[1,2,3†*], Aron Szekely[1,4†], Andrea Guido[1,2,5], Michele Gelfand[6], Jered Abernathy[7], Gizem Arikan[8], Zeynep Aycan[9,10], Shweta Bankar[11], Davide Barrera[4,12], Dana Basnight-Brown[13], Anabel Belaus[14,15], Elizaveta Berezina[16], Sheyla Blumen[17], Paweł Boski[18], Huyen Thi Thu Bui[19], Juan Camilo Cárdenas[20], Đorđe Čekrlija[21], Mícheál de Barra[22], Piyanjali de Zoysa[23], Angela Dorrough[24], Jan B. Engelmann[25], Hyun Euh[26], Susann Fiedler[27], Olivia Foster-Gimbel[28], Gonçalo Freitas[29], Marta Fülöp[30,31], Ragna B. Gardarsdottir[32], Colin Mathew Hugues D. Gill[16, 33], Andreas Glöckner[24], Sylvie Graf[34], Ani Grigoryan[35], Katarzyna Growiec[18], Hirofumi Hashimoto[36], Tim Hopthrow[37], Martina Hřebíčková[34], Hirotaka Imada[38], Yoshio Kamijo[39], Hansika Kapoor[40], Yoshihisa Kashima[41], Narine Khachatryan[35], Natalia Kharchenko[42], Diana León[43], Lisa M. Leslie[28], Yang Li[44], Kadi Liik[45], Marco Tullio Liuzza[46], Angela T. Maitner[47], Pavan Mamidi[11], Michele McArdle[8], Imed Medhioub[48], Maria Luisa Mendes Teixeira[49], Sari Mentser[50], Francisco Morales[51], Jayanth Narayanan[52], Kohei Nitta[53], Ravit Nussinson[54,55], Nneoma G. Onyedire[56], Ike E. Onyishi[56], Evgeny Osin[57], Seniha Özden[9], Penny Panagiotopoulou[58], Oleksandr Pereverziev[59], Lorena R. Perez-Floriano[60], Anna-Maija Pirttilä-Backman[61], Marianna Pogosyan[62], Jana Raver[63], Cecilia Reyna[14], Ricardo Borges Rodrigues[64], Sara Romanò[12], Pedro P. Romero[65,66], Inari Sakki[60], Angel Sánchez[67,68], Sara Sherbaji[47, 69], Brent Simpson[7], Lorenzo Spadoni[70], Eftychia Stamkou[71], Giovanni A. Travaglino[38], Paul A. M. Van Lange[73], Fiona Fira Winata[74], Rizqy Amelia Zein[74], Qing-peng Zhang[75], Kimmo Eriksson[2,76,77]

*Correspondence to: giulia.andrighetto@istc.cnr.it

†Equal contributions.

Fig. S1.

Changes in outcomes (Wave 2 - Wave 1) for hand washing norms (only COVID-relevant items). Change in appropriateness items is computed by scaling the average change in each country to the maximum possible change on the used scale in the survey. Hence, such an index may take values from -1 to +1. Red and black dots depict sampled cities. Indonesia is not included in Hand Washing Appropriateness data because of a mistake in the survey translation.

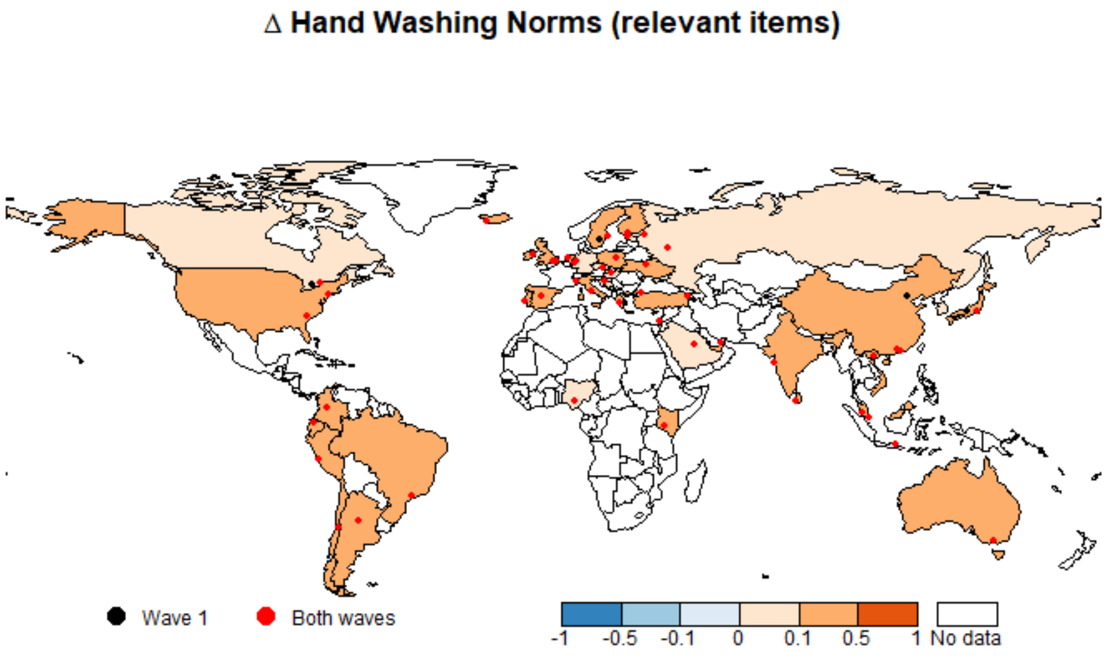

Fig. S2
Change in the Tightness-Looseness scores for each country after the emergence of the COVID-19 pandemic. Dots depicts the sum of fixed and random effects from a multilevel model of TL scores reported in Table S2 (Model 2). Bars report 95% CIs of the total effect. The solid vertical line reports the fixed effect estimate of *Wave 2*.

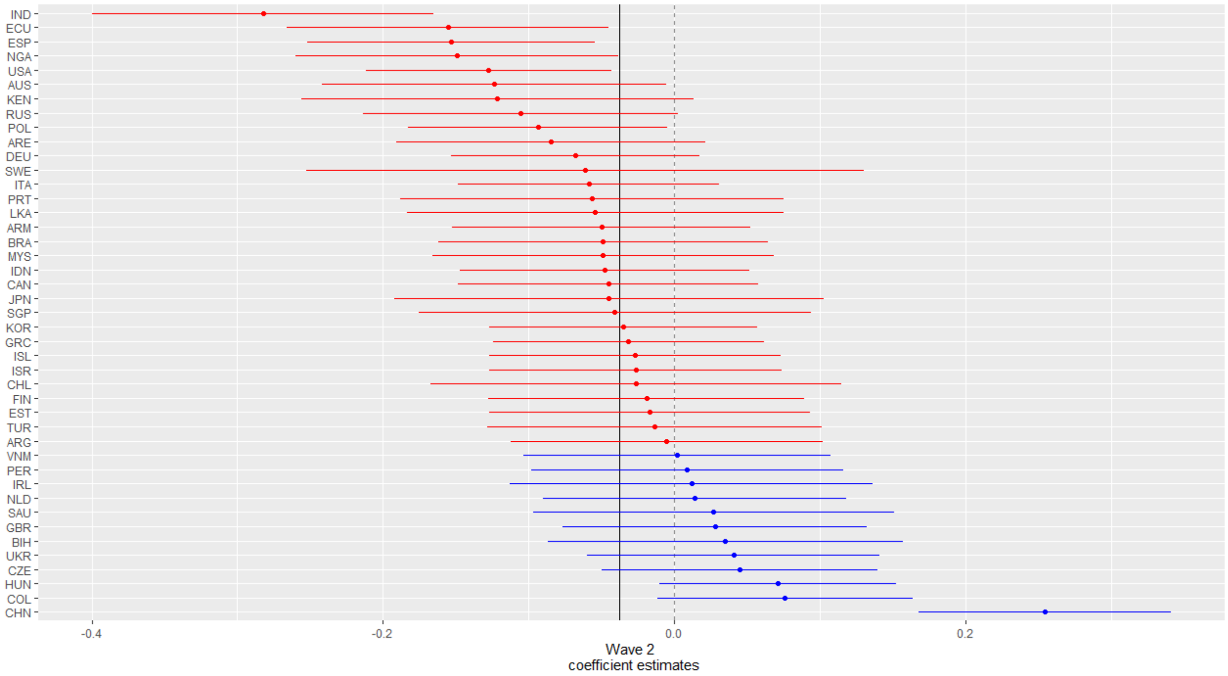

Fig. S3

Change in situation-specific norms for each country after the emergence of the COVID-19 pandemic. Dots depicts the sum of fixed and random effects from a multilevel model of TL scores reported in Table S4 (Model 2). Bars report 95% CIs of the total effect. The solid vertical line reports the fixed effect estimate of *Wave 2*.

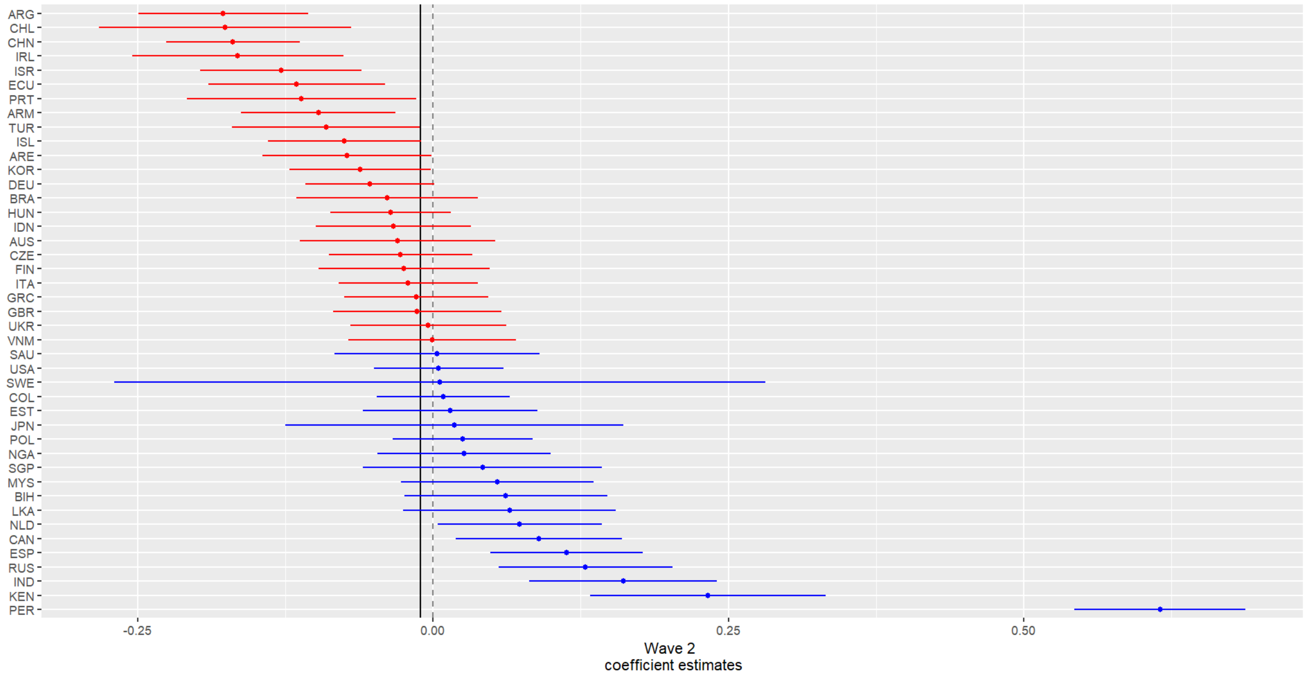

Fig. S4
Change in Metanorms (all items) for each country after the emergence of the COVID-19 pandemic. Dots depicts the sum of fixed and random effects from a multilevel model of Metanorms reported in Table S6 (Model 2). Bars shows 95% CIs. The solid vertical line reports the fixed effect estimate of *Wave 2*.

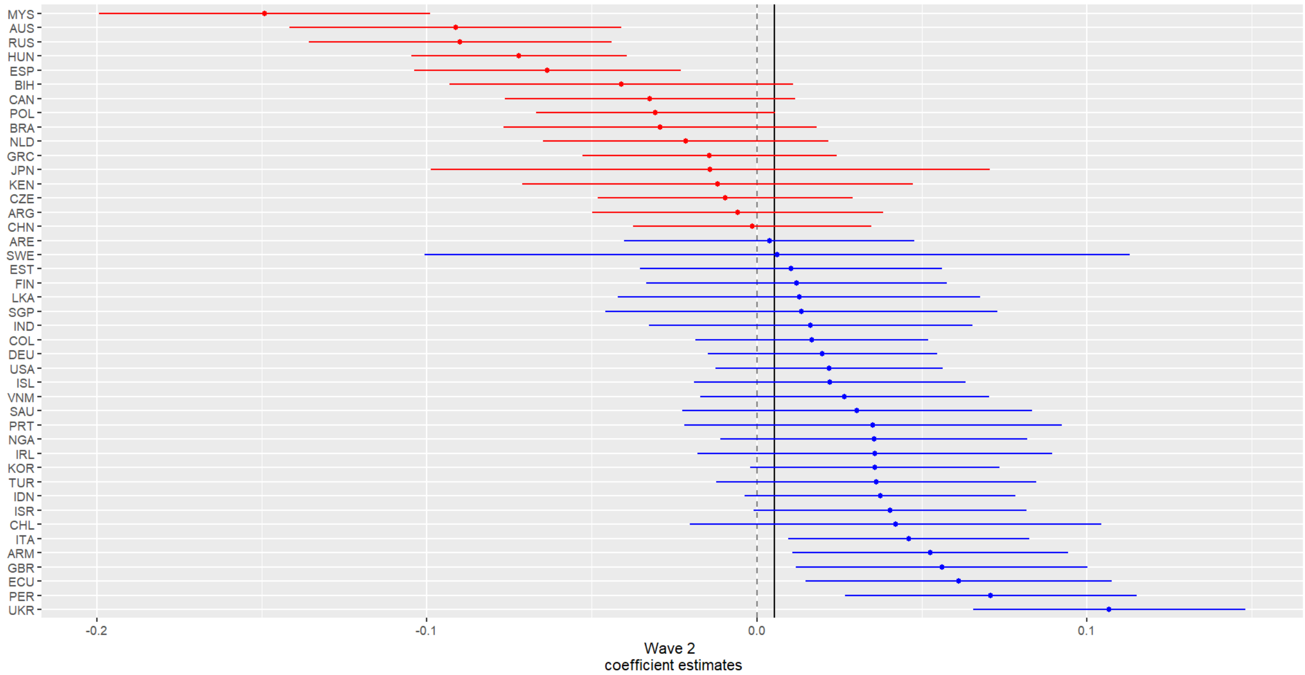

Fig. S5
Change in punishing frequency scores for each country after the emergence of the COVID-19 pandemic. Dots depicts the sum of fixed and random effects from a multilevel model of punishing frequency reported in Table S8 (Model 2). Bars shows 95% CIs. The solid vertical line reports the fixed effect estimate of *Wave 2*.

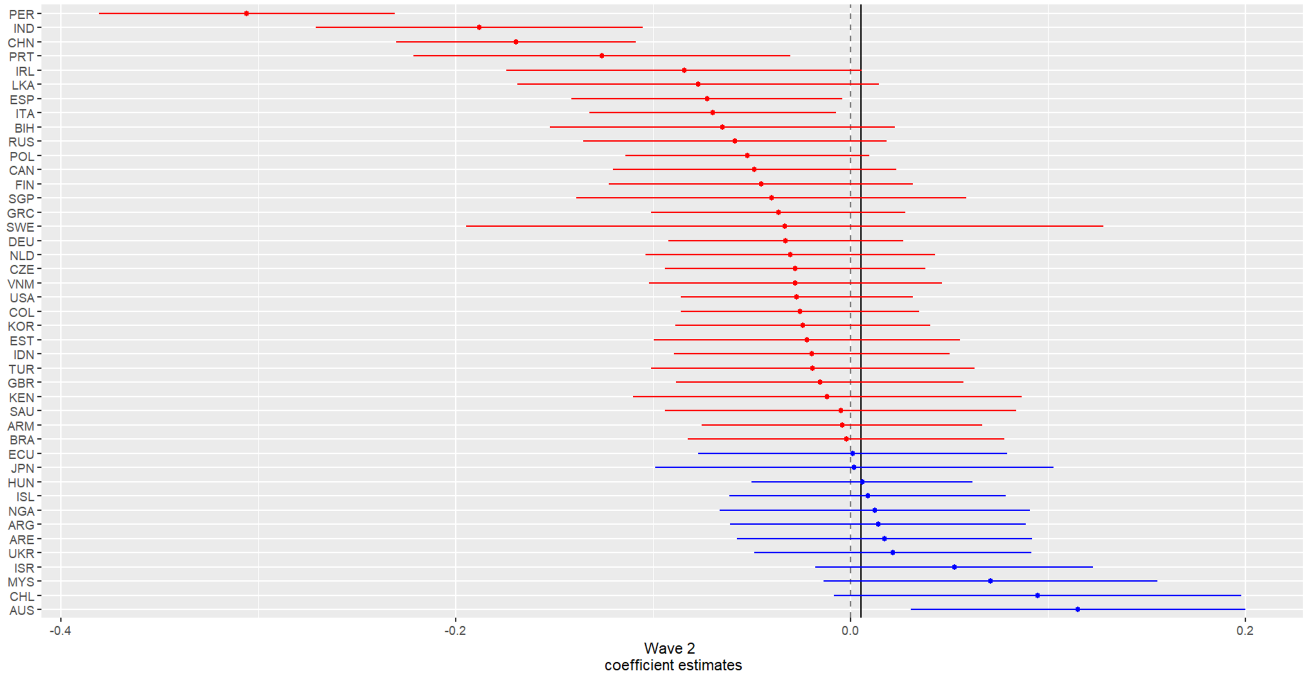

Fig. S6

Change in Hand Washing appropriateness scores for each country after the emergence of the COVID-19 pandemic. Dots depicts the sum of fixed and random effects from a multilevel model of Hand Washing norms (sum of ticked scenarios) reported in Table S10 (Model 2). Bars shows 95% CIs. The solid vertical line reports the fixed effect estimate of *Wave 2*.

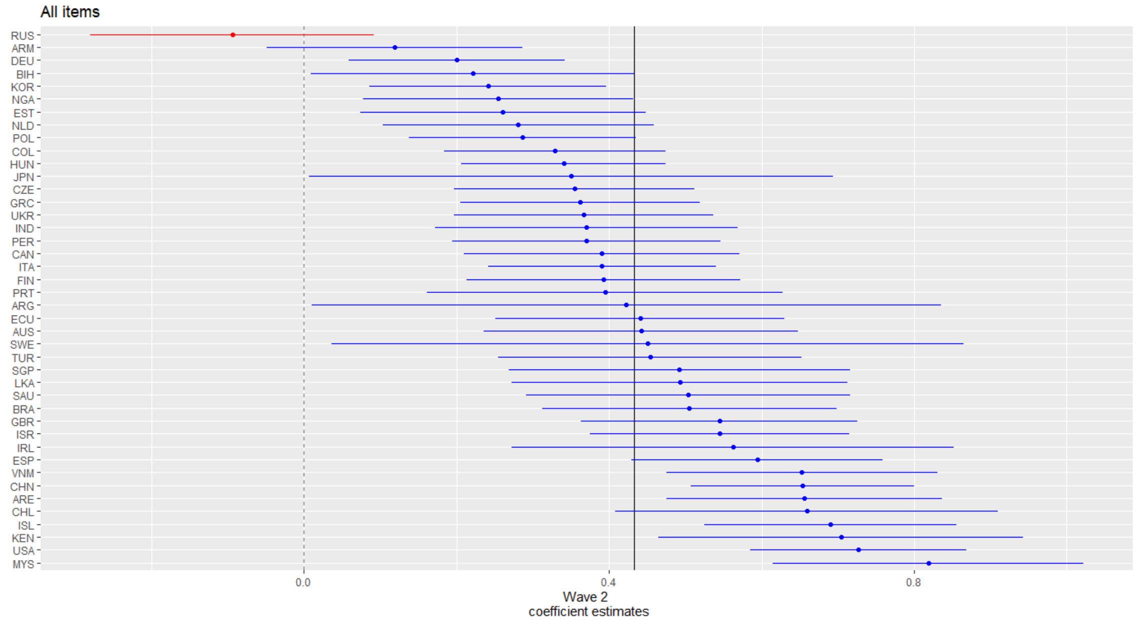

Fig. S7
On top, correlation between countries Tightness-Looseness scores (mean centered) across waves (panels A-B). At the bottom, evolution of scores over the three waves (panel C).

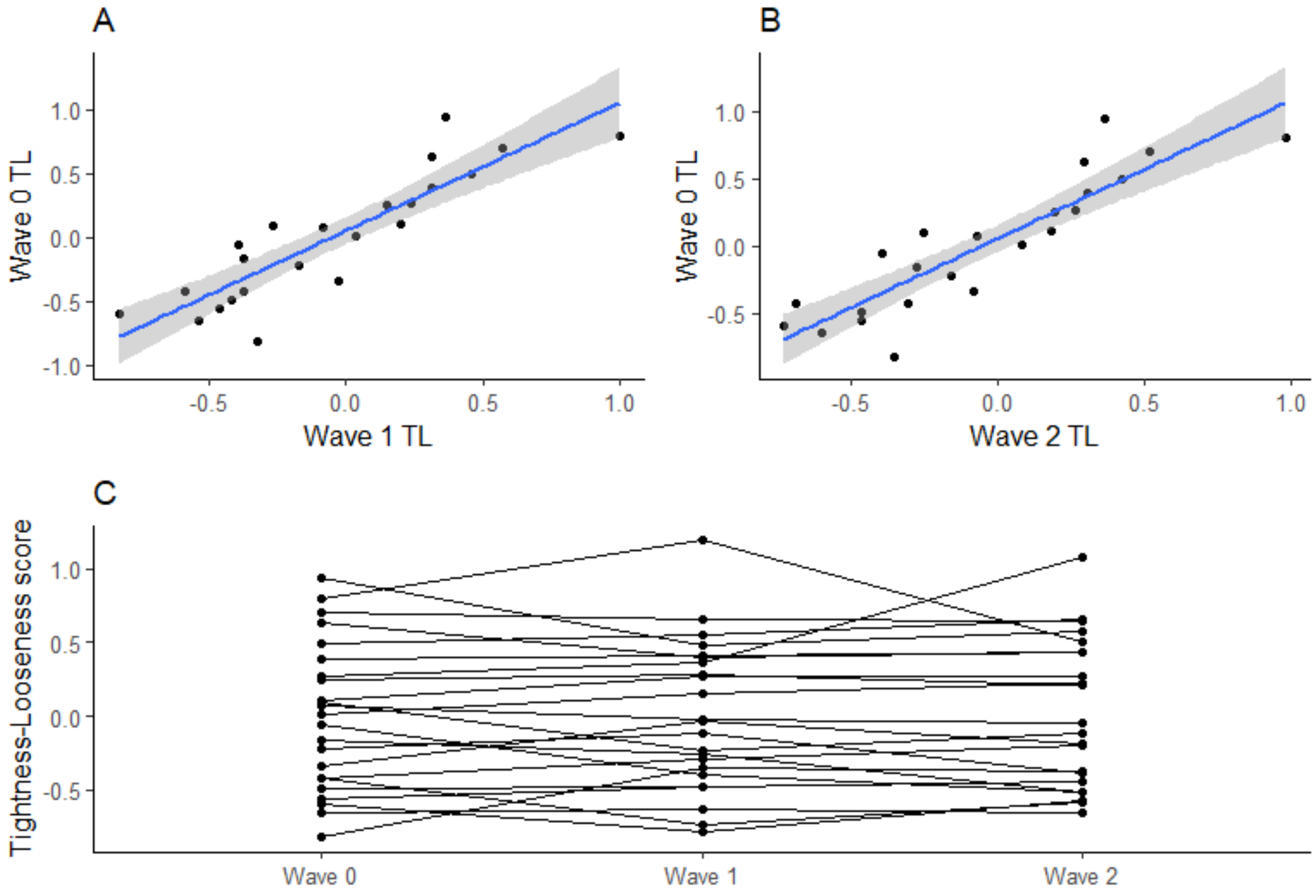

Notes: country-level data from Wave 0 are taken from Gelfand et al. 2011. In the analysis showed in panels A-C, we mean centre tightness-looseness in all three waves and standardize the scores (i.e. divide by the SD). This allows us to identify if countries that were tight in Wave 0, relative to other countries in Wave 0, remain tight in Wave 1 relative to the other countries in Wave 1 or *vice versa* and to detect any meaningful trends.

Table S1
Descriptive statistics of tightness-looseness scores, % of males and students in our samples, and average age by country.

		Sample Size		*Tightness Looseness scores*		*% Males*		*% Students*		*Age*	
**Country (ISO Code)**	**Location**	**Wave 1**	**Wave 2**	**Wave 1**	**Wave 2**	**Wave 1**	**Wave 2**	**Wave 1**	**Wave 2**	**Wave 1**	**Wave 2**
ARE	Sharjah	306	286	2.36	2.26	35.20	34.62	100.00	100.00	20.01	19.91
ARG	Cordoba	451	243	1.52	1.48	30.96	15.23	46.56	26.97	25.71	49.85
ARM	Yerevan	369	390	2.14	2.12	33.79	38.76	67.48	64.87	24.22	24.42
AUS	Melbourne	257	194	1.87	1.73	23.35	18.04	100.00	100.00	19.64	19.70
BIH	Banja Luka	244	173	1.59	1.68	50.21	33.92	100.00	100.00	21.19	22.67
BRA	Sao Paolo	277	419	1.62	1.59	44.77	42.48	67.87	66.83	31.36	40.01
CAN	Kingston	210	460	1.65	1.63	44.29	43.79	100.00	100.00	19.79	19.84
CAN	Toronto	223	-	1.98	-	21.62	-	100.00	-	20.78	-
CHL	Santiago	125	116	1.67	1.65	57.60	56.90	100.00	100.00	20.89	21.03
CHN	Beijing	409	-	2.10	-	27.21	-	71.15	-	22.72	-
CHN	Guangzhou	305	93	2.11	2.28	30.90	41.30	71.80	100.00	24.37	19.98
CHN	Shenzhen	343	386	2.05	2.43	27.30	58.09	100.00	100.00	20.17	20.44
COL	Bogota	341	895	1.48	1.58	39.59	40.22	67.74	70.39	25.46	24.84
CZE	Brno	386	450	1.55	1.61	22.66	23.33	68.13	64.22	28.32	30.28
DEU	Cologne	302	203	2.04	1.90	42.19	30.54	33.11	100.00	25.79	29.35
DEU	Hagen	372	222	2.03	2.04	21.29	25.68	0.00	0.00	31.46	31.14
ECU	Quito	286	244	1.80	1.59	37.54	37.30	84.27	86.89	22.45	23.21
ESP	Madrid	329	430	1.74	1.56	55.62	50.47	61.40	59.07	28.19	31.07
EST	Tallin	290	259	1.54	1.49	25.17	30.50	76.21	47.88	31.42	33.83
FIN	Helsinki	256	369	1.70	1.67	16.93	15.76	89.45	81.03	30.92	32.55
GBR	Canterbury	316	79	1.75	1.90	17.72	22.78	61.08	100.00	26.20	20.92
GBR	London	107	315	1.78	1.74	11.21	34.71	100.00	36.19	19.16	30.29
GRC	Athens	163	-	1.63	-	42.33	-	90.18	-	27.09	-

GRC	Patra	336	522	1.76	1.73	25.07	19.00	65.48	70.11	27.35	25.50
HUN	Budapest	452	843	1.45	1.52	20.13	16.07	100.00	60.62	24.47	29.02
IDN	Surabaya	319	396	2.35	2.29	18.81	25.00	69.59	65.66	22.55	23.16
IND	Mumbai	260	224	2.54	2.10	12.84	42.15	95.00	100.00	19.69	22.06
IRL	Dublin	242	146	1.76	1.84	41.49	45.89	19.42	100.00	22.17	21.77
ISL	Reykjavik	469	305	1.97	1.95	22.60	20.39	78.89	75.08	30.58	31.23
ISR	Raanana	334	350	1.62	1.60	39.82	32.18	67.66	60.00	27.78	28.40
ITA	Rome	188	277	1.79	1.77	55.32	48.01	100.00	100.00	22.25	24.40
ITA	Turin	207	278	1.94	1.84	27.18	34.89	100.00	100.00	23.44	24.60
JPN	Hikone	304	-	2.20	-	38.82	-	100.00	-	19.66	-
JPN	Kanagawa	259	-	2.00	-	54.44	-	100.00	-	20.14	-
JPN	Tokyo	-	494	-	2.06	-	60.29	-	100.00	-	18.97
KEN	Nairobi	190	113	2.36	2.21	50.53	25.66	100.00	100.00	22.01	27.73
KOR	Seoul	369	497	2.10	2.06	44.17	42.05	66.40	56.94	27.12	29.51
LKA	Colombo	191	170	2.49	2.45	37.89	46.75	100.00	100.00	23.19	23.15
MYS	Kuala Lumpur	344	164	2.15	2.13	45.64	29.63	69.48	85.37	25.06	22.64
NGA	Nsukka	284	416	2.37	2.18	40.00	40.98	63.03	66.83	27.82	24.97
NLD	Amsterdam	278	349	1.48	1.52	44.96	31.90	100.00	100.00	21.78	21.81
PER	Lima	261	423	1.68	1.72	32.57	45.84	45.98	49.88	32.43	27.74
POL	Warsaw	553	417	1.67	1.56	29.22	29.47	43.94	46.52	35.24	35.49
PRT	Lisbon	143	159	2.04	1.94	11.19	25.32	100.00	100.00	24.31	25.68
RUS	Moscow	271	227	1.58	1.45	21.40	12.39	100.00	100.00	23.04	20.68
RUS	St Petersburg	124	16	1.51	1.24	24.39	0.00	100.00	100.00	21.88	24.50
SAU	Riyhad	320	147	2.43	2.52	71.84	55.17	69.06	77.55	24.85	25.06
SGP	Singapore	201	157	2.25	2.17	31.34	41.03	100.00	100.00	21.96	21.43
SWE	Linkoping	163	-	2.22	-	45.40	-	100.00	-	24.75	-
SWE	Stockholm	40	152	2.08	1.96	47.50	47.37	100.00	100.00	37.38	NA
TUR	Istanbul	228	232	2.19	2.18	16.67	28.02	100.00	100.00	21.59	23.35

UKR	Kiev	315	564	1.69	1.63	29.62	39.61	67.94	47.70	29.23	31.58
USA	Columbia	457	203	1.86	1.76	22.20	21.18	100.00	100.00	19.44	19.98
USA	New York	210	274	1.71	1.53	25.24	21.17	100.00	100.00	21.89	22.89
VNM	Hanoi	466	245	2.25	2.27	22.94	26.23	100.00	100.00	18.76	19.48
Totals and averages		15445	14986	1.90	1.81	32.68	34.30	77.46	75.35	24.91	26.48

Notes: To assess sample comparability across waves, we conducted several tests comparing age, gender balance and student status at the country and, where possible, at the city level. Results show no statistical difference across waves in these controls. Exact data on participants' *Age* is not available for our Sweden (Stockholm) site of Wave 2. Yet, we collected participants' age range for this sample.

Table S2

Multilevel regression models of TL scores. Model (1) considers varying intercepts at the country and location level. Model (2) includes also varying slopes at the country level of the predictor Wave 2. Model (3) considers student participants only while Model (4) considers our non-student samples. Model (5) also includes country-level covariates to control for COVID-19 related variables such as number of cases and deaths (standardized). Data on deaths and cases are from Our World in Data (November 2020).

	**TL scores**														
		**(1)**			**(2)**			**(3)**			**(4)**			**(5)**	
Predictors	*Estimates*	*CI*	*p*	*Estimates*	*CI*	*p*	*Estimates*	*CI*	*p*	*Estimates*	*CI*	*p*	*Estimates*	*CI*	*p*
Constant	1.94	1.84 – 2.04	<0.001	1.94	1.84 – 2.05	<0.001	2.03	1.93 – 2.13	<0.001	1.81	1.68 – 1.94	<0.001	1.94	1.84 – 2.04	<0.001
Wave 2	-0.03	-0.05 – -0.01	0.003	-0.04	-0.07 – -0.00	0.042	-0.04	-0.06 – -0.02	<0.001	0.02	-0.02 – 0.06	0.295	-0.03	-0.05 – -0.01	0.003
Age	-0.00	-0.00 – -0.00	0.021	-0.00	-0.00 – -0.00	0.032	-0.00	-0.00 – -0.00	0.002	-0.00	-0.00 – 0.00	0.890	-0.00	-0.00 – -0.00	0.021
Male	-0.09	-0.11 – -0.07	<0.001	-0.09	-0.11 – -0.07	<0.001	-0.11	-0.13 – -0.09	<0.001	-0.07	-0.11 – -0.03	0.001	-0.09	-0.11 – -0.07	<0.001
Student	0.04	0.01 – 0.07	0.005	0.04	0.02 – 0.07	0.003							0.04	0.01 – 0.07	0.005
Covid Cases (sd)													-0.01	-0.14 – 0.13	0.942
Covid Deaths (sd)													-0.04	-0.18 – 0.09	0.538
							**Random Effects**								
σ^2		0.57			0.57			0.55			0.63			0.57	
τ_{00}		0.01_{Location}			0.01_{Location}			0.01_{Location}			0.00_{Location}			0.01_{Location}	
		0.08_{Country}			0.09_{Country}			0.09_{Country}			0.08_{Country}			0.08_{Country}	
τ_{11}					$0.01_{\text{Country.Wave2}}$										

ρ_{01}		-0.28 Country			
ICC	0.14	0.14	0.15		0.14
N	43 Country	43 Country	43 Country	26 Country	43 Country
	55 Location	55 Location	54 Location		55 Location
Observations	28374	28374	21656	6718	28374
Marginal R^2 / Conditional R^2	0.004 / 0.140	0.005 / 0.147	0.005 / 0.152	0.002 / 0.114	0.008 / 0.146
AIC	64959.028	64897.932	48809.595	16055.046	64969.879

Notes: Columns CI report the 95% confidence interval; p values reported from two-sided t-tests. ANOVA test comparing models (1) and (2) provides indication for a significant increase in predicting accuracy upon inclusion of varying slopes at the country level for the variable Wave 2 Chisq. = 63.72, p<.001).

Table S3

OLS models of change in Tightness-looseness scores. Observations at the country level. Heteroskedasticity-robust errors.

	Change in Tightness-Looseness score														
	**(1)**			**(2)**			**(3)**			**(4)**			**(5)**		
Predictors	*Estimates*	*95% CI*	*p*	*Estimates*	*95% CI*	*p*	*Estimates*	*95% CI*	*p*	*Estimates*	*95% CI*	*p*	*Estimates*	*95% CI*	*p*
Constant	0.275	-0.099 – 0.648	0.144	0.294	-0.067 – 0.655	0.108	0.274	-0.084 – 0.632	0.130	0.349	-0.034 – 0.731	0.073	0.372	-0.024 – 0.767	0.065
Fear of COVID-19	-0.064	-0.138 – 0.010	0.088	-0.070	-0.142 – 0.002	0.055	-0.077	-0.152 – -0.002	0.043	-0.055	-0.135 – 0.025	0.173	-0.081	-0.157 – -0.005	0.037
Perceived Prevalence	-0.004	-0.010 – 0.001	0.098	-0.003	-0.009 – 0.003	0.341	-0.005	-0.011 – 0.001	0.096	-0.005	-0.010 – 0.001	0.078	-0.003	-0.010 – 0.003	0.306
Gov. Stringency	0.001	-0.001 – 0.002	0.430	-0.000	-0.002 – 0.002	0.942	0.001	-0.001 – 0.002	0.427	0.000	-0.001 – 0.002	0.717	-0.000	-0.002 – 0.001	0.721
Age				0.001	-0.003 – 0.006	0.549							0.001	-0.005 – 0.006	0.837
% Male							0.199	-0.085 – 0.484	0.164				0.234	-0.047 – 0.515	0.100
% Students										-0.100	-0.257 – 0.057	0.206	-0.072	-0.266 – 0.122	0.456
Observations	41			40			41			41			40		
R^2 / R^2 adjusted	0.122 / 0.051			0.101 / -0.002			0.158 / 0.064			0.149 / 0.054			0.165 / 0.014		

Notes: Columns CI report the 95% confidence interval; p values reported from two-sided t-tests. Two countries are excluded in all models: Armenia (missing governmental stringency data) and Peru (missing perceived prevalence and threat due to a survey error); Sweden is missing in models (2-3-5) because of missing Age variable in Wave 2.

Table S4
Multilevel regression models of situation-specific norms. Dependent variable is the appropriateness rating in a given scenario. Model (1) considers varying intercepts at the country, city and scenario level. Model (2) also includes varying slopes at the country level for the predictor Wave 2. Model (3) controls for deaths and cases (standardized).

	Appropriateness rating in a given scenario								
	(1)			(2)			(3)		
Predictors	*Estimates*	*CI*	*p*	*Estimates*	*CI*	*p*	*Estimates*	*CI*	*p*
Constant	1.08	0.58 – 1.57	<0.001	1.08	0.58 – 1.57	<0.001	1.08	0.58 – 1.58	<0.001
Wave 2	-0.02	-0.03 – -0.01	0.003	-0.01	-0.05 – 0.03	0.628	-0.02	-0.03 – -0.01	0.003
Age	0.00	-0.00 – 0.00	0.055	0.00	-0.00 – 0.00	0.111	0.00	-0.00 – 0.00	0.054
Male	0.07	0.06 – 0.08	<0.001	0.07	0.06 – 0.08	<0.001	0.07	0.06 – 0.08	<0.001
Student	0.04	0.03 – 0.06	<0.001	0.04	0.03 – 0.06	<0.001	0.04	0.03 – 0.06	<0.001
Deaths (sd)							-0.09	-0.22 – 0.04	0.185
Cases (sd)							-0.02	-0.15 – 0.11	0.757
**Random Effects**									
σ^2	0.97			0.97			0.97		
τ_{00}	0.00 $_{Location}$			0.00 $_{Location}$			0.00 $_{Location}$		
	0.02 $_{Country}$			0.02 $_{Country}$			0.02 $_{Country}$		
	0.32 $_{Scenario}$			0.32 $_{Scenario}$			0.32 $_{Scenario}$		
τ_{11}				0.02 $_{Country.Wave2}$					
ρ_{01}				-0.15 $_{Country}$					

ICC	0.26	0.26	0.26
N	43 Country	43 Country	43 Country
	55 Location	55 Location	55 Location
	5 Scenario	5 Scenario	5 Scenario
Observations	142536	142536	142536
Marginal R^2 / Conditional R^2	0.001 / 0.260	0.001 / 0.263	0.003 / 0.260
AIC	401099.268	400680.992	401105.737

Notes: Columns CI report the 95% confidence interval; p values reported from two-sided t-tests.

Table S5
Multilevel regression models of situation-specific norms, analyzed in each scenario separately. Dependent variables report the appropriateness of a norm-breaking behavior in a given scenario (stealing, playing music at a funeral, sleeping in a restaurant, singing in a library, or reading a newspaper at the cinema). All models use varying intercepts at the country and location level.

	**Steal**			**Music at Funeral**			**Sleep at Rest.**			**Sing Library**			**Newspaper Cinema**		
Predictors	*Estimates*	*95% CI*	*p*	*Estimates*	*95% CI*	*p*	*Estimates*	*95% CI*	*p*	*Estimates*	*95% CI*	*p*	*Estimates*	*95% CI*	*p*
Constant	0.92	0.84 – 1.01	<0.001	0.70	0.61 – 0.79	<0.001	1.65	1.55 – 1.75	<0.001	0.62	0.54 – 0.70	<0.001	2.01	1.91 – 2.12	<0.001
Wave 2	-0.07	-0.09 – -0.04	<0.001	0.07	0.04 – 0.09	<0.001	-0.04	-0.06 – -0.02	0.001	0.07	0.05 – 0.09	<0.001	-0.09	-0.11 – -0.06	<0.001
Age	-0.00	-0.01 – -0.00	<0.001	-0.00	-0.00 – 0.00	0.074	-0.00	-0.00 – -0.00	<0.001	0.00	-0.00 – 0.00	0.366	-0.00	-0.01 – -0.00	<0.001
Male	0.13	0.10 – 0.15	<0.001	0.10	0.08 – 0.13	<0.001	0.07	0.04 – 0.09	<0.001	0.01	-0.01 – 0.03	0.279	0.07	0.04 – 0.10	<0.001
Student	0.03	-0.01 – 0.06	0.154	-0.00	-0.04 – 0.04	0.998	0.01	-0.02 – 0.05	0.548	-0.03	-0.06 – 0.00	0.086	0.04	-0.00 – 0.08	0.062
**Random Effects**															
σ^2	0.91			0.92			0.90			0.71			1.23		
τ_{00}	0.01_{Location}			0.01_{Location}			0.01_{Location}			0.01_{Location}			0.00_{Location}		
	0.04_{Country}			0.04_{Country}			0.06_{Country}			0.04_{Country}			0.06_{Country}		
ICC	0.05			0.06			0.08			0.06			0.05		
N	43_{Country}			43_{Country}			43_{Country}			43_{Country}			43_{Country}		
	55_{Location}			55_{Location}			55_{Location}			55_{Location}			55_{Location}		
Observations	29212			29252			29246			29274			29226		
Marginal R^2 / Conditional R^2	0.007 / 0.060			0.004 / 0.059			0.003 / 0.080			0.002 / 0.060			0.005 / 0.057		
AIC	80427.711			80863.200			80063.533			73358.978			89239.502		

Notes: Columns CI report the 95% confidence interval; p values reported from two-sided t-tests.

Table S6

Multilevel model of Meta norm items (4 actions for each of the 5 scenarios). Dependent variable is the appropriateness rating in a given scenario. Model (1) considers varying intercepts at the Countries, city and scenario levels. Model (2) allows for varying slopes of predictor Wave 2 at the Countries level. Model (3) includes deaths and cases from COVID-19 (standardized).

	**Meta norms**								
		**(1)**			**(2)**			**(3)**	
Predictors	*Estimates*	*CI*	*p*	*Estimates*	*CI*	*p*	*Estimates*	*CI*	*p*
Constant	0.687	0.148 – 0.826	0.005	0.687	0.147 – 0.826	0.005	0.690	0.146 – 0.834	0.005
Wave 2	0.006	-0.001 – 0.013	0.120	0.005	-0.013 – 0.023	0.570	0.003	-0.004 – 0.010	0.364
Mean Appropriateness	0.720	0.712 – 0.728	<0.001	0.720	0.712 – 0.728	<0.001	0.721	0.713 – 0.729	<0.001
Age	0.002	0.002 – 0.003	<0.001	0.002	0.002 – 0.003	<0.001	0.002	0.002 – 0.003	<0.001
Male	0.055	0.047 – 0.062	<0.001	0.052	0.045 – 0.060	<0.001	0.052	0.044 – 0.059	<0.001
Student	-0.009	-0.020 – 0.001	0.088	-0.011	-0.022 – -0.000	0.048	-0.014	-0.024 – -0.003	0.012
Deaths (sd)							0.069	-0.030 – 0.167	0.171
Cases (sd)							-0.087	-0.187 – 0.014	0.091
**Random Effects**									
σ^2	1.446			1.445			1.433		
τ_{00}	0.002 Locations			0.003 Locations			0.002 Locations		
	0.012 Countries			0.016 Countries			0.010 Countries		
	0.147 Scenarios			0.147 Scenarios			0.152 Scenarios		
τ_{11}				0.003 Countries.Wave					
ρ_{01}				-0.530 Countries					
ICC	0.107			0.108			0.109		

N	43 Countries<br>55 Locations<br>5 Scenarios	43 Countries<br>55 Locations<br>5 Scenarios	41 Countries<br>53 Locations<br>5 Scenarios
Observations	484670	484670	462451
Marginal R^2 / Conditional R^2	0.063 / 0.164	0.063 / 0.164	0.063 / 0.165

Notes: Columns CI report the 95% confidence interval; p values reported from two-sided t-tests.

Table S7
Multilevel model of metanorm items. Dependent variable is the appropriateness of a certain punishment behavior in a given situation (see Material and Methods). Varying intercepts at the country, location and situation levels.

	Verbal Conf			Ostracism			Gossip			Non Action (Reversed)			Physical		
Predictors	*Estimates*	*CI*	*p*	*Estimates*	*CI*	*p*	*Estimates*	*CI*	*p*	*Estimates*	*CI*	*p*	*Estimates*	*CI*	*p*
Constant	0.48	-0.21 – 1.17	0.171	-1.02	-1.34 – -0.70	<0.001	-0.87	-0.98 – -0.76	<0.001	1.08	0.63 – 1.54	<0.001	-0.67	-0.82 – -0.52	<0.001
Wave 2	-0.00	-0.01 – 0.02	0.698	0.03	0.01 – 0.04	<0.001	-0.02	-0.03 – -0.01	<0.001	-0.01	-0.02 – 0.00	0.065	0.02	-0.01 – 0.05	0.125
Mean Appropriateness	0.94	0.93 – 0.96	<0.001	1.27	1.26 – 1.29	<0.001	1.49	1.48 – 1.50	<0.001	0.87	0.86 – 0.89	<0.001	0.91	0.88 – 0.94	<0.001
Age	-0.00	-0.00 – -0.00	0.001	0.00	0.00 – 0.00	<0.001	0.00	0.00 – 0.00	<0.001	-0.00	-0.00 – -0.00	<0.001	0.01	0.01 – 0.02	<0.001
Male	0.01	-0.01 – 0.04	0.185	-0.05	-0.07 – -0.03	<0.001	0.01	-0.01 – 0.02	0.339	0.01	-0.01 – 0.03	0.297	0.00	-0.04 – 0.04	0.927
Student	-0.04	-0.06 – -0.03	<0.001	0.08	0.07 – 0.10	<0.001	0.01	-0.00 – 0.02	0.104	-0.14	-0.15 – -0.12	<0.001	0.18	0.15 – 0.21	<0.001
**Random Effects**															
σ^2	1.26			1.07			0.76			1.05			1.27		
τ_{00}	0.00 $_{Location}$			0.01 $_{Location}$			0.00 $_{Location}$			0.00 $_{Location}$			0.01 $_{Location}$		
	0.04 $_{Country}$			0.04 $_{Country}$			0.04 $_{Country}$			0.03 $_{Country}$			0.12 $_{Country}$		
	0.43 $_{Situation}$			0.14 $_{Situation}$			0.01 $_{Situation}$			0.35 $_{Situation}$					
ICC	0.29			0.15			0.06			0.27			0.09		
N	43 $_{Country}$			43 $_{Country}$			43 $_{Country}$			43 $_{Country}$			43 $_{Country}$		
	55 $_{Location}$			55 $_{Location}$			55 $_{Location}$			55 $_{Location}$			55 $_{Location}$		
	5 $_{Situation}$			5 $_{Situation}$			5 $_{Situation}$			5 $_{Situation}$					
Observations	142550			142550			142550			142550			28510		
Marginal R^2 / Conditional R^2	0.089 / 0.357			0.208 / 0.310			0.351 / 0.396			0.105 / 0.281			0.116 / 0.196		
AIC	350639.145			329531.018			289316.823			329079.375			87926.939		

Notes: Columns CI report the 95% confidence interval; p values reported from two-sided t-tests.

Table S8

Multilevel model of Punishment frequency. Model (1) considers varying intercepts at the country, city and scenario level. Model (2) also includes varying slope at the country level. Model (3) controls also for deaths and cases (standardized).

	Punishment frequency								
	(1)			(2)			(3)		
Predictors	*Estimates*	*CI*	*p*	*Estimates*	*CI*	*p*	*Estimates*	*CI*	*p*
Constant	2.658	2.576 – 2.741	<0.001	2.657	2.568 – 2.745	<0.001	2.653	2.566 – 2.740	<0.001
Wave 2	-0.034	-0.047 – -0.022	<0.001	-0.031	-0.059 – -0.003	0.028	-0.035	-0.048 – -0.023	<0.001
Mean appropriateness	0.199	0.184 – 0.213	<0.001	0.198	0.183 – 0.212	<0.001	0.200	0.185 – 0.214	<0.001
Age	-0.002	-0.003 – -0.002	<0.001	-0.002	-0.003 – -0.002	<0.001	-0.002	-0.003 – -0.001	<0.001
Male	-0.096	-0.109 – -0.083	<0.001	-0.092	-0.105 – -0.079	<0.001	-0.099	-0.113 – -0.086	<0.001
Student	0.047	0.028 – 0.065	<0.001	0.050	0.030 – 0.069	<0.001	0.050	0.031 – 0.069	<0.001
Deaths (sd)							0.048	-0.099 – 0.194	0.522
Cases (sd)							-0.018	-0.167 – 0.131	0.814
**Random Effects**									
σ^2	0.774			0.773			0.767		
τ_{00}	0.003 $_{Location}$			0.002 $_{Location}$			0.003 $_{Location}$		
	0.022 $_{Country}$			0.033 $_{Country}$			0.024 $_{Country}$		
	0.002 $_{Scenario}$			0.002 $_{Scenario}$			0.002 $_{Scenario}$		
τ_{11}				0.007 $_{Country.Wave}$					

ρ_{01}		-0.576 $_{Country}$	
ICC	0.033	0.035	0.036
N	3 $_{Scenario}$	3 $_{Scenario}$	3 $_{Scenario}$
	43 $_{Country}$	43 $_{Country}$	41 $_{Country}$
	55 $_{Location}$	55 $_{Location}$	53 $_{Location}$
Observations	85495	85495	81577
Marginal R^2 / Conditional R^2	0.015 / 0.047	0.014 / 0.049	0.015 / 0.051

Notes: Columns CI report the 95% confidence interval; p values reported from two-sided t-tests.

Table S9
Multilevel models of punishment frequency for each item separately. Models include varying intercepts at the country and location level.

	Confront			Gossip			Avoid		
Predictors	*Estimates*	*CI*	*p*	*Estimates*	*CI*	*p*	*Estimates*	*CI*	*p*
Constant	3.043	2.946 – 3.140	<0.001	2.667	2.566 – 2.768	<0.001	2.268	2.155 – 2.382	<0.001
Wave 2	-0.021	-0.041 – -0.002	0.035	-0.091	-0.112 – -0.070	<0.001	0.011	-0.012 – 0.034	0.335
Mean appropriateness	-0.047	-0.070 – -0.024	<0.001	0.349	0.325 – 0.373	<0.001	0.292	0.266 – 0.318	<0.001
Age	0.002	0.001 – 0.003	0.005	-0.010	-0.011 – -0.009	<0.001	0.001	-0.000 – 0.002	0.163
Male	0.002	-0.019 – 0.022	0.878	-0.185	-0.206 – -0.163	<0.001	-0.105	-0.128 – -0.081	<0.001
Student	0.073	0.043 – 0.103	<0.001	0.038	0.006 – 0.069	0.019	0.029	-0.005 – 0.063	0.098
**Random Effects**									
σ^2	0.666			0.718			0.839		
τ_{00}	0.001 $_{Location}$			0.006 $_{Location}$			0.008 $_{Location}$		
	0.038 $_{Country}$			0.037 $_{Country}$			0.053 $_{Country}$		
ICC	0.056			0.055			0.068		
N	43 $_{Country}$			43 $_{Country}$			43 $_{Country}$		
	55 $_{Location}$			55 $_{Location}$			55 $_{Location}$		
Observations	28505			28500			28490		
Marginal R^2 / Conditional R^2	0.002 / 0.057			0.059 / 0.111			0.020 / 0.087		

Notes: Columns CI report the 95% confidence interval; p values reported from two-sided t-tests.

Table S10
OLS regression models with heteroskedasticity-robust errors. Dependent variables are changes in responses to each frequency of punishment questions.

	Change in all items			Change in Confronting			Change in Gossiping			Change in Avoiding		
Predictors	*Estimates*	*CI*	*p*	*Estimates*	*CI*	*p*	*Estimates*	*CI*	*p*	*Estimates*	*CI*	*p*
Constant	0.064	-0.195 – 0.322	0.621	-0.250	-0.691 – 0.190	0.256	0.338	-0.289 – 0.964	0.281	0.104	-0.238 – 0.445	0.541
Fear of COVID-19	-0.019	-0.062 – 0.024	0.377	0.039	-0.031 – 0.109	0.267	-0.139	-0.261 – -0.016	0.028	0.042	-0.034 – 0.119	0.267
Perceived Prevalence	0.000	-0.004 – 0.004	0.956	0.002	-0.003 – 0.008	0.379	-0.000	-0.008 – 0.007	0.913	-0.002	-0.008 – 0.005	0.632
Gov. Stringency	-0.001	-0.002 – 0.001	0.314	-0.002	-0.004 – 0.000	0.099	-0.001	-0.005 – 0.003	0.584	0.001	-0.002 – 0.003	0.589
Age	-0.000	-0.003 – 0.003	0.947	0.003	-0.003 – 0.009	0.346	0.002	-0.005 – 0.009	0.540	-0.005	-0.009 – -0.001	0.022
Male	0.186	-0.052 – 0.424	0.122	0.092	-0.210 – 0.394	0.540	0.561	0.037 – 1.085	0.037	-0.095	-0.424 – 0.234	0.561
Student	-0.022	-0.145 – 0.102	0.725	0.056	-0.114 – 0.226	0.505	0.025	-0.194 – 0.243	0.821	-0.144	-0.341 – 0.053	0.146
Observations	40			40			40			40		
R^2 / R^2 adjusted	0.070 / -0.099			0.113 / -0.048			0.186 / 0.038			0.086 / -0.080		

Notes: Columns CI report the 95% confidence interval; p values reported from two-sided t-tests.

Table S11
Multilevel linear regression models. Model (1) reports the results of a model including varying intercepts at the country and location level. Model (2) also includes varying slopes at the country level for the variable *Wave 2*. The dependent variable of each model is the number of situations in which a respondent considers appropriate to wash hands (from 0 to 6).

Hand Washing Norms (number of situations ticked by each participant)									
		(1)			(2)			(3)	
Predictors	*Estimates*	*CI*	*p*	*Estimates*	*CI*	*p*	*Estimates*	*CI*	*p*
Constant	3.714	3.577 – 3.851	<0.001	3.703	3.540 – 3.866	<0.001	3.718	3.579 – 3.856	<0.001
Wave 2	0.420	0.390 – 0.450	<0.001	0.433	0.361 – 0.506	<0.001	0.420	0.390 – 0.450	<0.001
Age	-0.030	-0.077 – 0.016	0.200	-0.032	-0.079 – 0.014	0.175	-0.030	-0.076 – 0.016	0.205
Male	0.002	-0.000 – 0.004	0.087	0.002	-0.000 – 0.004	0.111	0.002	-0.000 – 0.004	0.084
Student	-0.177	-0.208 – -0.146	<0.001	-0.179	-0.209 – -0.148	<0.001	-0.177	-0.208 – -0.146	<0.001
Cases (sd)							-0.084	-0.242 – 0.075	0.303
Deaths (sd)							0.038	-0.119 – 0.196	0.632
**Random Effects**									
σ^2	1.45			1.45			1.45		
τ_{00}	0.06 $_{Locations}$			0.05 $_{Locations}$			0.06 $_{Locations}$		
	0.06 $_{Countries}$			0.15 $_{Countries}$			0.07 $_{Countries}$		
τ_{11}				0.04 $_{Countries.Wave}$					
ρ_{01}				-0.72 $_{Countries}$					
ICC	0.08			0.09			0.08		
N	42 $_{Countries}$			42 $_{Countries}$			42 $_{Countries}$		
	53 $_{Locations}$			53 $_{Locations}$			53 $_{Locations}$		
Observations	28139			28139			28139		
Marginal R^2 / Conditional R^2	0.032 / 0.109			0.033 / 0.118			0.034 / 0.113		
AIC	90634.894			90530.702			90644.863		

Notes: Columns CI report the 95% confidence interval; p values reported from two-sided t-tests.

Table S12

Multilevel logit regression models of Hand Washing norms, for each possible situation. The dependent variables take value 1 if a participant ticked that particular situation in response to our item, 0 otherwise.

	**Before Meal**			**After Meal**			**After Defecating**			**After Urinating**			**After Shaking Hands**			**After Come Home**		
Predictors	*Odds Ratios*	*95% CI*	*p*	*Odds Ratios*	*95% CI*	*p*	*Odds Ratios*	*95% CI*	*p*	*Odds Ratios*	*95% CI*	*p*	*Odds Ratios*	*95% CI*	*p*	*Odds Ratios*	*95% CI*	*p*
Constant	7.31	5.49 – 9.73	<0.001	0.88	0.64 – 1.21	0.426	25.30	17.52 – 36.54	<0.001	15.97	11.78 – 21.65	<0.001	0.08	0.07 – 0.10	<0.001	1.12	0.83 – 1.50	0.455
Wave 2	1.26	1.16 – 1.36	<0.001	1.33	1.26 – 1.41	<0.001	1.00	0.90 – 1.11	0.933	1.14	1.03 – 1.27	0.010	2.74	2.58 – 2.91	<0.001	2.53	2.37 – 2.70	<0.001
Age	1.05	1.00 – 1.11	0.067	0.98	0.95 – 1.02	0.307	1.05	0.97 – 1.14	0.198	0.87	0.82 – 0.93	<0.001	1.05	1.01 – 1.09	0.007	1.11	1.06 – 1.17	<0.001
Male	1.04	0.92 – 1.18	0.517	0.93	0.86 – 1.01	0.077	0.86	0.73 – 1.02	0.078	0.95	0.82 – 1.10	0.482	0.91	0.83 – 0.99	0.038	1.04	0.93 – 1.15	0.496
Student	0.81	0.75 – 0.88	<0.001	0.91	0.86 – 0.97	0.001	0.78	0.70 – 0.87	<0.001	0.71	0.64 – 0.78	<0.001	0.86	0.81 – 0.92	<0.001	0.61	0.57 – 0.65	<0.001
**Random Effects**																		
σ^2	3.29			3.29			3.29			3.29			3.29			3.29		
τ_{00}	0.08 Locations			0.11 Locations			0.02 Locations			0.09 Locations			0.07 Locations			0.23 Locations		
	0.54 Countries			0.86 Countries			0.93 Countries			0.49 Countries			0.14 Countries			0.58 Countries		
ICC	0.16			0.23			0.23			0.15			0.06			0.20		
N	42 Countries			42 Countries			42 Countries			42 Countries			42 Countries			42 Countries		
	53 Locations			53 Locations			53 Locations			53 Locations			53 Locations			53 Locations		
Observations	28139			28139			28139			28139			28139			28139		
Marginal R^2 / Conditional R^2	0.006 / 0.163			0.005 / 0.233			0.005 / 0.229			0.012 / 0.160			0.071 / 0.126			0.064 / 0.248		
AIC	18130.910			34970.998			11770.826			12788.422			30204.498			26462.178		

Notes: Columns CI report the 95% confidence interval; p values reported from two-sided t-tests.

Table S13

OLS regression of change in handwashing items. Errors robust to heteroskedasticity. Dependent variables are changes in number of ticking per each handwashing situation. Predicted variable "*Relevant Items"* includes the average of items mostly related to the spread of the pandemics (i.e., *After Shaking Hands* and *After Come Home*). Predicted variable *"Not Relevant Items"* includes the average of non-directly related items (i.e., *Before meal, After meal, After defecating, After urinating*).

	**All Items**			**Not Relevant Items**			**Relevant Items**			**After Shaking Hands**			**After Come Home**		
Predictors	*Estimates*	*95% CI*	*p*	*Estimates*	*95% CI*	*p*	*Estimates*	*95% CI*	*p*	*Estimates*	*95% CI*	*p*	*Estimates*	*95% CI*	*p*
Constant	-0.176	-0.331 – -0.021	0.027	-0.106	-0.240 – 0.027	0.113	-0.322	-0.602 – -0.041	0.026	-0.322	-0.588 – -0.057	0.019	-0.317	-0.686 – 0.052	0.090
Fear of COVID-19	0.040	0.004 – 0.076	0.032	0.014	-0.016 – 0.044	0.353	0.092	0.035 – 0.148	0.002	0.107	0.054 – 0.161	<0.001	0.075	0.002 – 0.148	0.044
Perceived Prevalence	0.002	-0.000 – 0.005	0.085	0.001	-0.001 – 0.004	0.162	0.004	-0.001 – 0.008	0.086	0.004	0.001 – 0.008	0.015	0.003	-0.003 – 0.010	0.283
Gov. Stringency	-0.000	-0.001 – 0.001	0.723	-0.000	-0.001 – 0.001	0.828	-0.000	-0.002 – 0.001	0.640	0.000	-0.001 – 0.002	0.647	-0.001	-0.003 – 0.001	0.355
Age	0.002	-0.000 – 0.004	0.113	0.001	-0.000 – 0.003	0.091	0.002	-0.001 – 0.006	0.236	-0.000	-0.004 – 0.003	0.923	0.004	-0.001 – 0.009	0.079
Male	-0.037	-0.141 – 0.067	0.476	0.007	-0.094 – 0.109	0.884	-0.126	-0.333 – 0.081	0.225	-0.142	-0.341 – 0.058	0.158	-0.111	-0.390 – 0.169	0.427
Student	0.016	-0.072 – 0.104	0.717	0.011	-0.062 – 0.083	0.763	0.027	-0.106 – 0.160	0.679	-0.044	-0.162 – 0.074	0.455	0.098	-0.091 – 0.286	0.299
Observations	40			40			40			40			40		
R^2 / R^2 adjusted	0.270 / 0.137			0.168 / 0.016			0.328 / 0.205			0.467 / 0.370			0.191 / 0.044		

Notes: Columns CI report the 95% confidence interval; p values reported from two-sided t-tests. Three countries were excluded from these analyses: Armenia because of missing Governmental Stringency data in the Oxford Government Tracker; Peru for missing data about Fear of COVID-19 and prevalence (due to error in the survey); Indonesia for missing data about hand-washing appropriateness scores across situations (due to survey translation error).

Table S14

Test of parallel trends using regression models. Errors robust to heteroskedasticity. Data from three waves at the country level.

	TL-scores											
	(1)			(2)			(3)			(4)		
Predictors	*Estimates*	*CI*	*p*	*Estimates*	*CI*	*p*	*Estimates*	*CI*	*p*	*Estimates*	*CI*	*p*
Constant	-2.380	-5.278 – 0.518	0.106	0.284	-0.406 – 0.973	0.414	0.036	-0.206 – 0.277	0.769	0.013	-0.288 – 0.314	0.931
Wave 1	-0.140	-4.497 – 4.217	0.949	0.089	-0.859 – 1.038	0.851	0.030	-0.302 – 0.362	0.856	0.015	-0.400 – 0.429	0.944
Wave 2	0.251	-3.805 – 4.308	0.902	0.391	-0.546 – 1.328	0.408	0.051	-0.280 – 0.381	0.761	0.045	-0.371 – 0.460	0.831
Fear of COVID-19	0.541	-0.133 – 1.214	0.114									
Fear of COVID-19 * Wave 1	0.032	-0.977 – 1.041	0.950									
Fear of COVID-19 * Wave 2	-0.057	-0.996 – 0.882	0.904									
Perceived Prevalence				-0.013	-0.043 – 0.018	0.410						
Perceived Prevalence * Wave 1				-0.004	-0.047 – 0.039	0.853						
Perceived Prevalence * Wave 2				-0.017	-0.058 – 0.023	0.388						
Covid Deaths							-0.001	-0.002 – 0.000	0.188			
Covid Deaths * Wave 1							-0.001	-0.002 – 0.001	0.496			

Covid Deaths * Wave 2							-0.001	-0.002 – 0.000	0.194			
Covid Cases										-0.000	-0.000 – 0.000	0.855
Covid Cases * Wave 1										-0.000	-0.000 – 0.000	0.886
Covid Cases * Wave 2										-0.000	-0.000 – 0.000	0.688
Observations	69			69			69			69		
R^2 / R^2 adjusted	0.138 / 0.070			0.068 / -0.006			0.066 / -0.008			0.007 / -0.072		

Notes: Columns CI report the 95% confidence interval; p values reported from two-sided t-tests.

Supplementary Note 1

We use the data from Gelfand et al. (2011) to check for parallel trends in tightness-looseness pre-COVID 19 but do not use this to identify a relevant SESOI for tightness-looseness as it is not feasible. This was done for the following reasons.

The data reported in Gelfand et al. 2011 (Wave 0) are not available in their raw form and only after within-subject standardization. This procedure, which we also use (Wave 2) and so does Eriksson et al. (2021; Wave 1), aims to account for cultural tendencies for responding to surveys in certain ways (e.g. systematically more/less extreme), and is done by calculating the mean for each person's responses to a set of questions that do not concern tightness-looseness in the survey and then subtracting that mean from each tightness-looseness item. Since our survey, and that conducted by Gelfand et al. 2011, differ in the non-tightness-looseness items used for standardization, there will be systematic differences between the adjusted values of the countries reported in Gelfand et al. 2011 and the values that we find. This can be directly seen by comparing the country scores reported in Gelfand et al. 2011 (Table 1) and our country scores (Table S1). Because of these differences, we cannot meaningfully compare the changes in absolute values between Wave 0 and Wave 1, preventing us from identifying a SESOI based on average changes in tightness-looseness of countries.

Nevertheless, we are able to use the Wave 0 data to check for parallel trends in the tightness-looseness of countries pre-COVID. Specifically, in the analysis on p. 11 (also Figure S7, Table S14), we check whether the countries that later (between Wave 1 and 2) are more/less affected by COVID-19 already systematically differ in their trends beforehand. To check for this, we mean centre tightness-looseness in all three waves and standardize the scores (i.e. divide by the SD). This allows us to identify if countries that were tight in Wave 0, relative to other countries in Wave 0, remain tight in Wave 1 relative to the other countries in Wave 1 or *vice versa* and to detect any meaningful trends. This is sufficient to check for parallel trends since if the assumption is violated, countries that were less/more affected by COVID would systematically deviate in their relative tightness-looseness across the waves. Yet since we standardise and mean centre tightness-looseness, average change in tightness looseness between Wave 0 and 1 cannot be calculated meaningfully (i.e. overall average change is 0).

There are two further relevant points. First, even though the absolute values differ between Wave 0 and Wave 1, the Wave 0 country-level values correlate very strongly with both Wave 1 (r=0.89) and Wave 2 (r=0.88). Second, because we use exactly the same items to standardize tightness-looseness as Eriksson et al. (2021; Wave 1), we are able to compare meaningfully Wave 1 to Wave 2. Moreover, comparing unstandardized tightness-looseness scores across waves 1 and 2 leads to the same findings.

References

Eriksson, Kimmo, Pontus Strimling, Michele Gelfand, Junhui Wu, Jered Abernathy, Charity S. Akotia, Alisher Aldashev, Per A. Andersson, Giulia Andrighetto, Adote Anum, Gizem Arikan, Zeynep Aycan, Fatemeh Bagherian, Davide Barrera, Dana Basnight-

Brown, Birzhan Batkeyev, Anabel Belaus, Elizaveta Berezina, Marie Björnstjerna, Sheyla Blumen, Paweł Boski, Fouad Bou Zeineddine, Inna Bovina, Bui Thi Thu Huyen, Juan-Camilo Cardenas, Đorđe Čekrlija, Hoon-Seok Choi, Carlos C. Contreras-Ibáñez, Rui Costa-Lopes, Mícheál de Barra, Piyanjali de Zoysa, Angela Dorrough, Nikolay Dvoryanchikov, Anja Eller, Jan B. Engelmann, Hyun Euh, Xia Fang, Susann Fiedler, Olivia A. Foster-Gimbel, Márta Fülöp, Ragna B. Gardarsdottir, C. M. Hew D. Gill, Andreas Glöckner, Sylvie Graf, Ani Grigoryan, Vladimir Gritskov, Katarzyna Growiec, Peter Halama, Andree Hartanto, Tim Hopthrow, Martina Hřebíčková, Dzintra Iliško, Hirotaka Imada, Hansika Kapoor, Kerry Kawakami, Narine Khachatryan, Natalia Kharchenko, Ninetta Khoury, Toko Kiyonari, Michal Kohút, Lê Thuỳ Linh, Lisa M. Leslie, Yang Li, Norman P. Li, Zhuo Li, Kadi Liik, Angela T. Maitner, Bernardo Manhique, Harry Manley, Imed Medhioub, Sari Mentser, Linda Mohammed, Pegah Nejat, Orlando Nipassa, Ravit Nussinson, Nneoma G. Onyedire, Ike E. Onyishi, Seniha Özden, Penny Panagiotopoulou, Lorena R. Perez-Floriano, Minna S. Persson, Mpho Pheko, Anna-Maija Pirttilä-Backman, Marianna Pogosyan, Jana Raver, Cecilia Reyna, Ricardo Borges Rodrigues, Sara Romanò, Pedro P. Romero, Inari Sakki, Alvaro San Martin, Sara Sherbaji, Hiroshi Shimizu, Brent Simpson, Erna Szabo, Kosuke Takemura, Hassan Tieffi, Maria Luisa Mendes Teixeira, Napoj Thanomkul, Habib Tiliouine, Giovanni A. Travaglino, Yannis Tsirbas, Richard Wan, Sita Widodo, Rizqy Zein, Qing-peng Zhang, Lina Zirganou-Kazolea, and Paul A. M. Van Lange. 2021. "Perceptions of the Appropriate Response to Norm Violation in 57 Societies." *Nature Communications* 12(1):1481. doi: 10.1038/s41467-021-21602-9.

Gelfand, Michele J., Jana L. Raver, Lisa Nishii, Lisa M. Leslie, Janetta Lun, Beng Chong Lim, Lili Duan, Assaf Almaliach, Soon Ang, Jakobina Arnadottir, Zeynep Aycan, Klaus Boehnke, Pawel Boski, Rosa Cabecinhas, Darius Chan, Jagdeep Chhokar, Alessia D'Amato, Montse Ferrer, Iris C. Fischlmayr, Ronald Fischer, Marta Fülöp, James Georgas, Emiko S. Kashima, Yoshishima Kashima, Kibum Kim, Alain Lempereur, Patricia Marquez, Rozhan Othman, Bert Overlaet, Penny Panagiotopoulou, Karl Peltzer, Lorena R. Perez-Florizno, Larisa Ponomarenko, Anu Realo, Vidar Schei, Manfred Schmitt, Peter B. Smith, Nazar Soomro, Erna Szabo, Nalinee Taveesin, Midori Toyama, Evert Van de Vliert, Naharika Vohra, Colleen Ward, and Susumu Yamaguchi. 2011. "Differences between Tight and Loose Cultures: A 33-Nation Study." *Science* 332(6033):1100–1104. doi: 10.1126/science.1197754.
