# Supplementary material for: Changes in social norms during the early stages of the COVID-19 pandemic across 43 countries
Source: Nat Commun. 2024 Feb 16;15:1436. doi: 10.1038/s41467-024-44999-5 (PMC10873354; doi:10.1038/s41467-024-44999-5)
Supplement: Supplementary file 2 — Reporting Summary [file 41467_2024_44999_MOESM2_ESM.pdf]

nature portfolio

Corresponding author(s): Giulia Andrighetto

Last updated by author(s): Dec 21, 2023

Reporting Summary

Nature Portfolio wishes to improve the reproducibility of the work that we publish. This form provides structure for consistency and transparency in reporting. For further information on Nature Portfolio policies, see our Editorial Policies and the Editorial Policy Checklist.

Statistics

For all statistical analyses, confirm that the following items are present in the figure legend, table legend, main text, or Methods section.

n/a	Confirmed	
☐	☒	The exact sample size (n) for each experimental group/condition, given as a discrete number and unit of measurement
☐	☒	A statement on whether measurements were taken from distinct samples or whether the same sample was measured repeatedly
☐	☒	The statistical test(s) used AND whether they are one- or two-sided<br>*Only common tests should be described solely by name; describe more complex techniques in the Methods section.*
☐	☒	A description of all covariates tested
☐	☒	A description of any assumptions or corrections, such as tests of normality and adjustment for multiple comparisons
☐	☒	A full description of the statistical parameters including central tendency (e.g. means) or other basic estimates (e.g. regression coefficient) AND variation (e.g. standard deviation) or associated estimates of uncertainty (e.g. confidence intervals)
☐	☒	For null hypothesis testing, the test statistic (e.g. F, t, r) with confidence intervals, effect sizes, degrees of freedom and P value noted<br>*Give P values as exact values whenever suitable.*
☒	☐	For Bayesian analysis, information on the choice of priors and Markov chain Monte Carlo settings
☐	☒	For hierarchical and complex designs, identification of the appropriate level for tests and full reporting of outcomes
☐	☒	Estimates of effect sizes (e.g. Cohen's d, Pearson's r), indicating how they were calculated

Our web collection on statistics for biologists contains articles on many of the points above.

Software and code

Policy information about availability of computer code

Data collection	Data were gather using Qualtrics (https://www.qualtrics.com). Survey code available at https://osf.io/stkfr/.
Data analysis	Data were analyzed using R v.4.1.1. Analysis code is available at https://osf.io/stkfr/.

For manuscripts utilizing custom algorithms or software that are central to the research but not yet described in published literature, software must be made available to editors and reviewers. We strongly encourage code deposition in a community repository (e.g. GitHub). See the Nature Portfolio guidelines for submitting code & software for further information.

Data

Policy information about availability of data

All manuscripts must include a data availability statement. This statement should provide the following information, where applicable:

- Accession codes, unique identifiers, or web links for publicly available datasets
- A description of any restrictions on data availability
- For clinical datasets or third party data, please ensure that the statement adheres to our policy

The data generated in this study have been deposited in the Open Science Framework (https://osf.io/stkfr/). Non-experimental data included in our datasets (i.e., intensity of government response to COVID-19 is the Stringency Index, COVID-19 deaths and cases per million) are taken from the Oxford COVID-19 Government Response Tracker21 and Our World in Data35 (downloaded November 2020).

Research involving human participants, their data, or biological material

Policy information about studies with human participants or human data. See also policy information about sex, gender (identity/presentation), and sexual orientation and race, ethnicity and racism.

Reporting on sex and gender	We considered gender in the design of the study and respondents could self-report their gender. We control for gender in all analyses.
Reporting on race, ethnicity, or other socially relevant groupings	We did not record information on race/ethnicity.
Population characteristics	See the section "research sample" below.
Recruitment	Participants were invited to participate in an "international study of social norms" using a range of methods including email invitations, social media, in online classes, existing experimental laboratory subject pools, and using survey organisations.<br><br>It is difficult to see how any self-selection bias could drive our main findings about the effect of COVID-19 on norms. This is particularly the case since we exploit variation in disease intensity and implement a difference-in-differences like design to test for plausible mechanisms.
Ethics oversight	All participants gave their informed consent and we complied with all relevant ethical regulations. Approval of the study protocol was obtained from ethics committees and institutional review boards where required including for the University of Melbourne (Australia), Queen's University at Kingston (Canada), Universidad de los Andes (Colombia), Institute of Psychology, Czech Academy of Sciences (Czech Republic), Universidad San Francisco de Quito (Ecuador), United Research Ethics Committee of Psychology (Hungary), Monk Prayogshala (India), Trinity College Dublin (Ireland), Open University of Israel (Israel), LUISS University (Italy), United States International University - Africa (Kenya), Sunway University (Malaysia), University of Amsterdam (Netherlands), SWPS University of Social Sciences and Humanities (Poland), Universidade de Lisboa (Portugal), National University of Singapore (Singapore), University of Colombo (Sri Lanka), Koc University (Turkey), American University of Sharjah (United Arab Emirates), Brunel University London (United Kingdom), University of Kent (United Kingdom), University of South Carolina (United States of America), and New York University (United States of America). Ethical approval was not sought in countries where the approval received for the study conducted in Wave 1 was considered sufficient or where local legislation did not require ethical approval in the first place.

Note that full information on the approval of the study protocol must also be provided in the manuscript.

Field-specific reporting

Please select the one below that is the best fit for your research. If you are not sure, read the appropriate sections before making your selection.

☐ Life sciences ☒ Behavioural & social sciences ☐ Ecological, evolutionary & environmental sciences

For a reference copy of the document with all sections, see nature.com/documents/nr-reporting-summary-flat.pdf

Behavioural & social sciences study design

All studies must disclose on these points even when the disclosure is negative.

Study description	We designed a quantitative study to understand how disease leads to norm change across societies and contexts. Specifically, we combine data from a survey collected between April-December 2019 (Eriksson et al. 2021 ; Wave 1) prior to the pandemic with a repeat of the same survey, in the same countries and sampled from the same populations, that we conducted in March-July 2020 (Wave 2) during the first months of COVID-19.

Research sample	We sampled populations in as many and as geographically diverse countries as we could. This yielded samples in 55 cities in 43 countries across the world. Samples were not representative (see sampling strategy below). For comparability of samples across waves and among countries, we set out to collect data from at least approximately 200 college students in a major city in each country, which was achieved in all countries. The sample size goal of 200 was set to match or surpass the sample sizes achieved in two closely related prior studies (Eriksson et al. 2021; Gelfand et al. 2011).<br><br>We use data collected by Eriksson et al. 2021 (https://doi.org/10.1038/s41467-021-21602-9) as the Wave 1, pre-COVID comparison, and test for differences relative to the data that we collect (Wave 2).<br><br>To assess the robustness of the country-level measures obtained from these samples, we complemented the main sampling strategy in two ways: we collected additional data from (1) non-student samples in 26 countries and (2) student samples from two or more cities in 11 countries. After excluding a small number of subjects who did not pass attention checks at the end of the survey and who reported an age under 18, we are left with 30,431 (students: n = 23,255; non-students: n = 7,176) participants. The gender composition was 33% males, 65% females, and 1% unknown. Gender was determined on self-report.<br><br>Non-experimental data included in our datasets (i.e., intensity of government response to COVID-19 is the Stringency Index, COVID-19 deaths and cases per million) are taken from the Oxford COVID-19 Government Response Tracker and Our World in Data (downloaded November 2020).
Sampling strategy	We used a convenience sampling strategy: participants were recruited using invitations via email, on social media, in class, face to face on campus, using public notices and flyers, and using survey organisations. This strategy was adopted for both Wave 1 and Wave 2; note in Wave 2 the in class and face to face recruitment did not occur. The sample size of at least 200 students per country was determined to match the prior closely related studies of Eriksson et al. 2021, who successfully measured how metanorms varies across countries, and Gelfand et al. 2011 who successfully studied how norms and tightness vary across countries.
Data collection	We used the surveys from Eriksson et al. 2021 that had been translated, using the independent translation and back-translation procedure, into 30 different languages. Additionally, we added a small number of questions to the end of the survey (e.g. to elicit fear of COVID-19 and perceived prevalence of COVID-19) and these were also translated and back translated. All data were collected online through Qualtrics. Researchers were were not present during survey completion and researchers were not blind to the study hypotheses.<br><br>In our analysis, we also use data from the Oxford Government Response Tracker (https://www.bsg.ox.ac.uk/research/research-projects/covid-19-government-response-tracker; Hale et al. 2021) and Our World in Data (https://ourworldindata.org/) deaths and cases for COVID-19 across countries.
Timing	Wave 1 data that we use were collected between April 2019 and December 2019. Wave 2 data were collected between March 2020 and July 2020.
Data exclusions	We excluded subjects who did not pass an attention check placed at the end of the survey (i.e. subjects had to click a specific item response). The total number of discarded observations because of failed attention check is minimal across waves relative to our sample size (197 in Wave 1, 202 in Wave 2). We additionally excluded subjects who declared an age under 18 (157 in Wave 1, 222 in Wave 2).
Non-participation	Because recruitment methods included open invitations (e.g. on social media and flyers) we have no data on the number of people who could have participated but chose not to.
Randomization	The participants were not allocated into experimental groups.

Reporting for specific materials, systems and methods

We require information from authors about some types of materials, experimental systems and methods used in many studies. Here, indicate whether each material, system or method listed is relevant to your study. If you are not sure if a list item applies to your research, read the appropriate section before selecting a response.

Materials & experimental systems

n/a	Involved in the study	
☒	☐	Antibodies
☒	☐	Eukaryotic cell lines
☒	☐	Palaeontology and archaeology
☒	☐	Animals and other organisms
☒	☐	Clinical data
☒	☐	Dual use research of concern
☒	☐	Plants

Methods

n/a	Involved in the study	
☒	☐	ChIP-seq
☒	☐	Flow cytometry
☒	☐	MRI-based neuroimaging

Plants

Seed stocks	*Report on the source of all seed stocks or other plant material used. If applicable, state the seed stock centre and catalogue number. If plant specimens were collected from the field, describe the collection location, date and sampling procedures.*
Novel plant genotypes	*Describe the methods by which all novel plant genotypes were produced. This includes those generated by transgenic approaches, gene editing, chemical/radiation-based mutagenesis and hybridization. For transgenic lines, describe the transformation method, the number of independent lines analyzed and the generation upon which experiments were performed. For gene-edited lines, describe the editor used, the endogenous sequence targeted for editing, the targeting guide RNA sequence (if applicable) and how the editor was applied.*
Authentication	*Describe any authentication procedures for each seed stock used or novel genotype generated. Describe any experiments used to assess the effect of a mutation and, where applicable, how potential secondary effects (e.g. second site T-DNA insertions, mosiacism, off-target gene editing) were examined.*
